# Genome-Wide Association Study and Candidate Gene Mining for Plant Height and Main Stem Node Number in Soybean from Northwest China

**DOI:** 10.3390/plants15111670

**Published:** 2026-05-29

**Authors:** Xudong Lu, Minglei Cheng, Yaqian Li, Lili Sun, Bingjie Niu, Min Wang, Bo Zhao, Lixiang Wang

**Affiliations:** 1 Shanxi Houji Laboratory, College of Agriculture, Shanxi Agricultural University, Jinzhong 030801, China; 20232128@stu.sxau.edu.cn (X.L.); 20235026@stu.sxau.edu.cn (M.C.); b20221024@stu.sxau.edu.cn (L.S.); 242005@sxau.edu.cn (B.N.); 2 College of Agriculture, Shanxi Agricultural University, Jinzhong 030801, China; 20232147@stu.sxau.edu.cn (Y.L.); wangmin@sxau.edu.cn (M.W.); 3 College of Resources and Environment, Shanxi Agricultural University, Jinzhong 030801, China

**Keywords:** soybean (*Glycine max* (L.) Merr.), plant height, main stem node number, GWAS, candidate gene

## Abstract

The Northwest soybean production region (covering Shanxi, Shaanxi, Gansu, Ningxia, Xinjiang, central and western Inner Mongolia and northern parts of Hebei) possesses vast cultivated land resources and advantageous light–temperature conditions, endowing soybean with substantial yield potential. In this study, two natural soybean populations originating from this region were used to systematically investigate the phenotypic variation in two important agronomic traits, plant height (PH) and main stem node number (NN). The results showed abundant genetic variation for both traits. Through genome-wide association analysis (GWAS) and employing a joint detection across multi-environments (control false positives), 5 SNPs significantly associated with PH and 18 SNPs significantly associated with NN were identified, among which four SNPs were detected associated with both traits. Candidate genes were further screened within the ±100 kb intervals flanking lead SNPs at association peaks. By integrating gene expression levels of different soybean tissues and their correlations with the phenotypes, two candidate genes associated with both PH and NN were determined. These findings provide a theoretical basis for the identification and utilization of soybean germplasm resources in Northwest China, and lay a solid foundation for breeding high-yield and high-quality soybean varieties through molecular breeding.

## 1. Introduction

Soybean (*Glycine max* (L.) Merr.) originated in China and is an important food and oil crop. The Northwest soybean production region in China has advantages including long sunshine duration, large diurnal temperature variation, and thermal resources, which provide natural conditions for high quality and high yield as well as substantial yield potential. However, this region has a temperate continental climate with challenges such as drought, low rainfall, and monotonous cropping patterns. The soybean planting area in this region accounts for less than 5% of the total grain crop area, and the average yield is below 1800 kg/ha. Therefore, there is an urgent need to overcome this production bottleneck via genetic improvement. Identifying genetic loci and candidate genes associated with important agronomic traits is significant for germplasm innovation, improving soybean self-sufficiency, and breeding high-quality, high-yield soybean varieties in Northwest China [1,2]. In recent years, genome-wide association study (GWAS) has become a powerful tool for dissecting the genetic basis of agronomic traits, provides theoretical foundations for soybean molecular breeding [3,4].

Soybean yield is collectively determined by key yield components, including the number of plants per unit area, pod number per plant, seed number per pod, and seed weight. Among these, the number of plants per unit area is significantly influenced by plant architecture-related traits, particularly plant height and main stem node number [5]. Notably, excessive plant height tends to induce lodging, thereby reducing soybean yield [6]. Main stem node number, however, not only shows a significantly positive correlation with both plant height and yield per unit area but also plays a more direct and crucial role in yield formation compared to plant height [7,8,9]. A reduction in main stem node number typically leads to a decrease in plant height, subsequently resulting in fewer pods per plant and ultimately lowering yield per plant [10]. Thus, different agronomic traits directly or indirectly affect soybean quality and yield. Moreover, these traits are susceptible to environmental conditions and genotype-by-environment interactions, increasing the complexity of genetic improvement [11]. Therefore, systematically dissecting the genetic mechanisms underlying soybean agronomic traits and identifying key genes controlling important traits such as plant height and main stem node number are of great significance for achieving high-yield breeding goals in soybean.

Traits such as plant height and main stem node number belong to plant architecture related traits [12]. Wang et al. [13] performed a GWAS on soybean plant height using 6.7 million SNPs and Indels, identified three QTLs on chromosomes 10, 18, and 19. Among these, the QTL on chromosome 19 was precisely co-localized with the previously confirmed plant height related gene *Dt1*, suggesting that the remaining QTLs may also be associated with plant height. In another study, Wang et al. [14] conducted association analysis using a recombinant inbred line population derived from ‘Dongnong L13’ and ‘Henong 60’, together with 455 germplasm accessions, and predicted two candidate genes associated with plant height, *Glyma.02G133000* and *Glyma.05G240600*, through pathway analysis and qRT-PCR validation. Furthermore, main stem node number directly affects soybean branch number and pod number, functioning as an important yield-related trait. Ding et al. [15] performed association analysis on 224 soybean core germplasm accessions using 1514 SNPs, detected 15 SNPs significantly associated with main stem node number, and identified *Glyma.12G142900* as a candidate gene.

However, existing studies on soybean GWAS have mostly focused on specific ecological regions or limited germplasm resources. Notably, GWAS analyses targeting mixed populations that integrate germplasm from the northwest production region of China with other soybean accessions remain relatively scarce. In this study, we used two mixed populations consisting primarily of Northwest germplasm but also including soybean germplasm from across China and other countries as experimental materials. GWAS was performed to identify SNP loci significantly associated with two key agronomic traits—plant height and main stem node number and to mine relevant candidate genes. This study is expected to provide a theoretical basis for molecular breeding of soybean. Furthermore, it is of great significance for the developing soybean varieties with desirable plant architecture, the guarantee of national food security, and the promoting sustainable agricultural development.

## 2. Results

### 2.1. Phenotypic Variation in Soybean Populations in Northwest China

**Figure 1:**
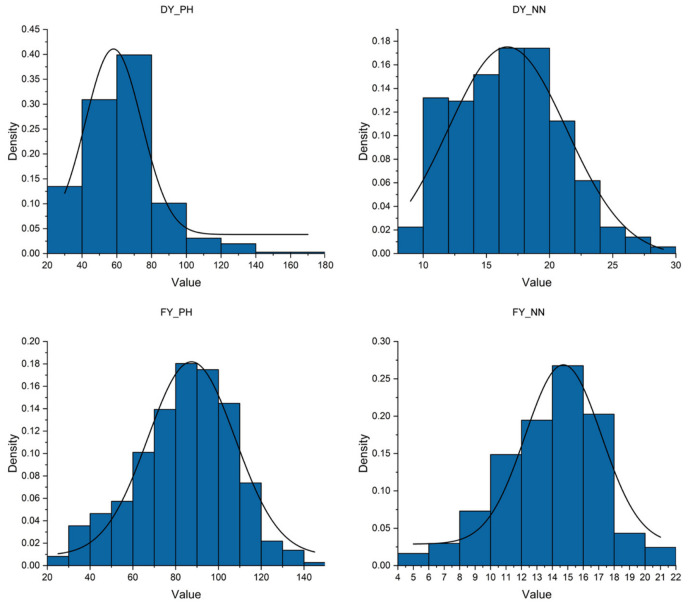
Frequency distribution of the phenotypic data. DY_PH: plant height in Dongyang; DY_NN: Main Stem Node Number in Dongyang; FY_PH: plant height in Fenyang; FY_NN: Main Stem Node Number in Fenyang. The *x*-axis represents the value range of different phenotypes, and the *y*-axis represents the probability density value. The black curve represents the result of the nonlinear curve fitting.

In this study, plant height and main stem node number were collected and analyzed from 372 accessions of a Northwest soybean natural population planted in 2024 at the experimental field of the Institute of Industrial Crops, Shanxi Agricultural University, in Fenyang City, Shanxi Province, and 384 accessions of another Northwest soybean natural population planted in 2025 at the Dongyang Experimental Base of Shanxi Agricultural University in Jinzhong City, Shanxi Province (Tables S1 and S2). Phenotypic analysis results showed that the average plant heights in Fenyang and Dongyang (Jinzhong) were 84.21 cm and 63.28 cm, respectively, with maximum values of 144.2 cm and 171.5 cm, and minimum values of 25 cm and 22.17 cm. For the main stem node numbers, the average values at the two locations were 13.79 and 16.67, with maximum values of 21.4 and 28.67 and minimum values of 5.6 and 8.67, respectively (Table 1). Collectively, plant height in Fenyang (FY_PH) was greater than that in Dongyang (DY_PH), whereas main stem node number in Fenyang (FY_NN) was lower than that in Dongyang (DY_NN). In addition, the phenotypic coefficients of variation for all traits at both locations exceeded 20% (Table 1), indicating abundant phenotypic variation in both plant height and main stem node number. Frequency distribution histograms were separately plotted for each trait at the two locations to visually display the data distributions. As shown in Figure 1, the phenotypic data for each trait were concentrated and approximately normally distributed, an essential characteristic that renders the datasets suitable for subsequent association analysis.

### 2.2. GWAS and Identification of Significant Associated Loci

**Figure 2:**
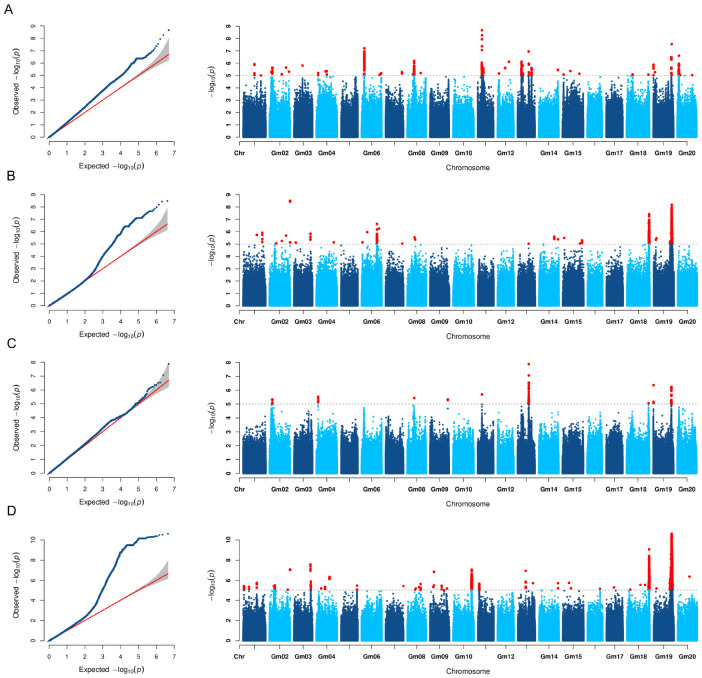
Q-Q plot and Manhattan plot of soybean height, main stem node number. (**A**) DY_PH. (**B**) FY_PH. (**C**) DY_NN. (**D**) FY_NN. The SNPs are represented by dots. (**A**–**D**) In the Q-Q plot, the *x*-axis represents the –log_10_(*p*) of the theoretical distribution, and the *y*-axis represents the –log_10_(*p*) of the actual observed distribution. If the data completely conform to the theoretical distribution, the points will be tightly distributed along the diagonal line. (**A**–**D**) In the Manhattan plot, the horizontal dashed line represents the −log_10_(*p*) ≥ 5. Red dots above the dashed line indicate SNPs significantly associated with the target trait.

Based on high-quality SNPs, GWAS was performed for soybean plant height and main stem node number at both locations (Figure 2). For each subfigure, the left panel presents the quantile-quantile (Q-Q) plot, which illustrates the distribution of observed −log_10_(*p*) values versus expected −log_10_(*p*) values under the null hypothesis of no association; the right panel displays the Manhattan plot, where the dashed line denotes the significance threshold. Using −log_10_(*p*) ≥ 5 as the significance threshold, SNPs with −log_10_(*p*) values above the dashed line were considered significantly associated with the respective trait. Both plots confirmed the presence of genome-wide loci significantly associated with each trait.

In total, 6324 SNPs tightly associated with plant architecture related traits were detected. Among these, 1186 SNPs were tightly associated with plant height in materials planted in Fenyang, and 445 SNPs were tightly associated with plant height in materials planted in the Dongyang. For main stem node number, 4620 SNPs were tightly associated in the Fenyang planted materials, and 73 SNPs were tightly associated in the Dongyang planted materials (Table S3).

To minimize the false positive rate, SNP markers detected at only a single location were removed, and only repeatedly detected SNPs were retained. Ultimately, 5 SNP markers significantly associated with soybean plant height and 18 SNP markers significantly associated with main stem node number were identified on chromosome 19 (Table 2 and Table 3). Notably, all SNPs significantly associated with plant height were also detected in the association results for main stem node number.

### 2.3. Analysis of Major Associated Loci and Candidate Intervals

To further identify candidate genes for plant height and main stem node number, the significance levels of different loci were compared by analyzing the significance of SNP association signals in Manhattan and Q-Q plots. The SNP locus Gm19:48168661 (Table 2), significantly associated with plant height, and the SNP locus Gm19:48211293 (Table 3), significantly associated with main stem node number, were selected as the major loci for each trait for further analysis.

**Figure 3:**
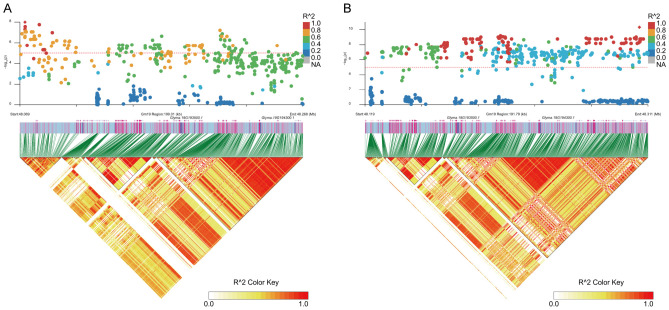
LD block plots of major loci. (**A**) Linkage disequilibrium analysis within the 100 kb upstream and downstream region of the Gm19:48168661 locus. (**B**) Linkage disequilibrium analysis within the 100 kb upstream and downstream region of the Gm19:48211293 locus. (**A**,**B**) The horizontal dashed line represents the −log(*p* value) ≥ 5. The red bars at the top indicate significant loci, the middle part shows gene distribution, and the bottom part reflects the LD intensity relationship between significant SNPs and other SNPs.

Linkage disequilibrium analysis was performed within the 100 kb upstream and downstream regions flanking each major locus using LD block plots. The results revealed linkage disequilibrium relationships among SNPs within these intervals (Figure 3). Based on the GWAS results, the 100 kb upstream and downstream regions flanking the physical positions of the major loci for plant height and main stem node number were defined as the candidate gene selection intervals. To improve screening efficiency, priority was given to candidate genes harboring non-synonymous mutations in exonic regions. Ultimately, 9 candidate genes associated with plant height and 11 candidate genes associated with main stem node number were obtained (Table 4 and Table 5). Notably, the candidate genes associated with main stem node number encompassed all candidate genes associated with plant height, suggesting that these two traits may be co-regulated by a subset of common genes.

Multiple candidate genes identified in this study have been previously reported to be associated with soybean plant architecture traits. Among them, *Glyma.19G192800* and *Glyma.19G194600* were identified by Song et al. as candidate genes for soybean vine growth habit traits [16]; *Glyma.19G192900* (*GmABCG39*), *Glyma.19G193400*, and *Glyma.19G193900* were predicted by Wang et al. as candidate genes associated with plant height and main stem node number under phosphorus treatment, and *GmABCG39* was also confirmed to positively regulate soybean plant height and main stem node number under phosphorus metabolism [17]; *Glyma.19G193100* was identified by Zhang et al. as a candidate gene associated with main stem node number [18]; *Glyma.19G194100* was identified by Chen et al. as a candidate gene associated with leaf morphology [19]; *Glyma.19G194900* (*GmBIC1c*) was confirmed by Mu et al. to regulate soybean plant height by promoting internode elongation and thereby controlling main stem node number [20]; *Glyma.19G194300* (*GmDt1*) was validated by Liu et al. as a key gene controlling soybean podding habit, and overexpression of *GmDt1* not only alters podding habit but also increases plant height and main stem node number [21]. Therefore, *GmABCG39* and *GmDt1* can be considered as effector genes associated with plant height, while *GmBIC1c* and *GmDt1* can be considered as effector genes associated with main stem node number.

**Figure 4:**
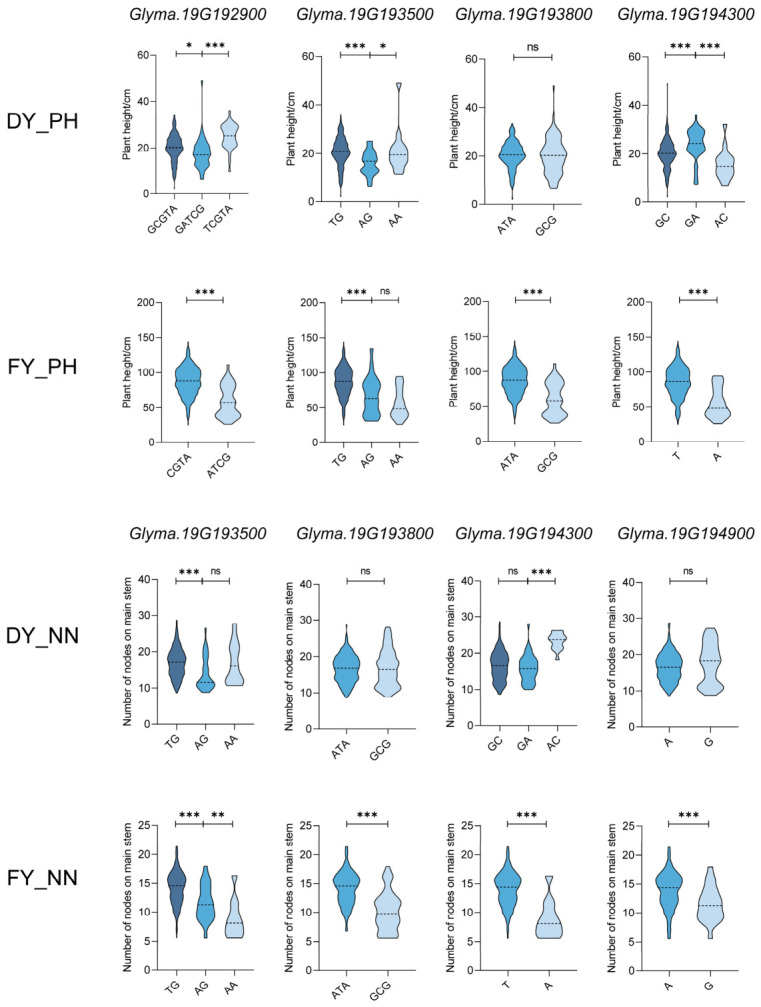
Correlation between candidate genes (with non-synonymous SNP mutations) and phenotypic traits. DY_PH: plant height in Dongyang; DY_NN: Main Stem Node Number in Dongyang; FY_PH: plant height in Fenyang; FY_NN: Main Stem Node Number in Fenyang. In the violin plot, the *x*-axis represents the types of base changes for different non-synonymous mutations, the *y*-axis represents different phenotypic data, and the center line represents the mean value. Asterisks indicate significant differences based on two-tailed Student’s *t*-test: ns: not significant; ***: significant at the 0.001 level; **: significant at the 0.01 level; *: significant at the 0.05 level.

**Figure 5:**
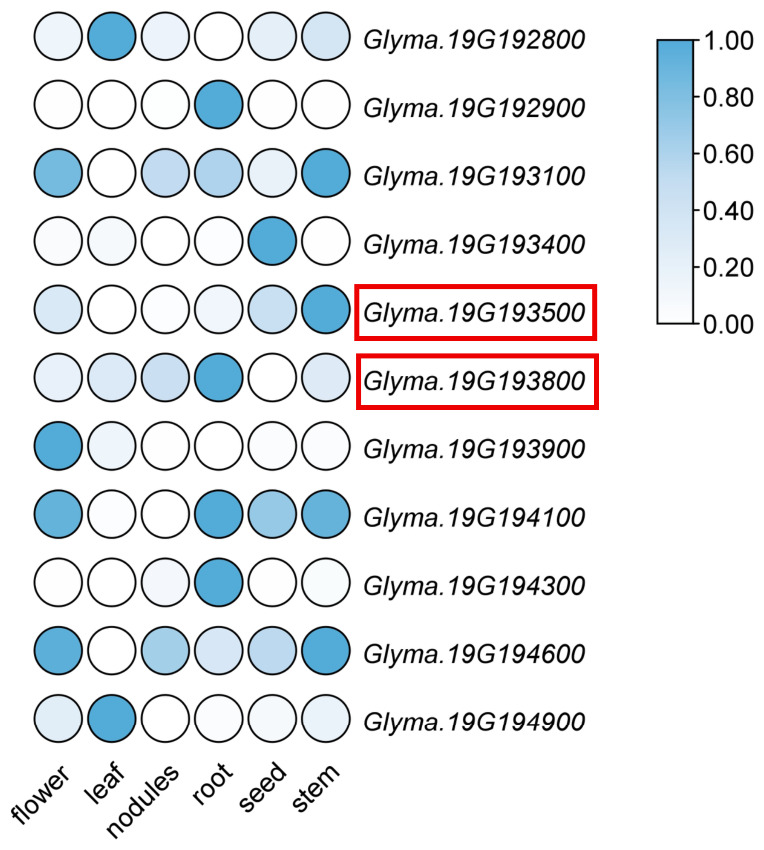
Candidate gene expression levels in different tissues. The heatmap was generated based on FPKM values from the Phytozome database and normalized by row. Blue indicates higher gene expression levels, while white indicates lower expression levels. The red box represents the filtered candidate genes.

For the candidate genes that have not yet been studied, *Glyma.19G193500* and *Glyma.19G193800*, as well as the genes for the relevant traits, we analyzed the correlation between their genotypes and the phenotypic traits. The results showed that *Glyma.19G193500* and *Glyma.19G193800*, together with the relevant effector genes, were significantly correlated with both plant height and main stem node number (Figure 4). Further comparison of the expression data of different genes across various tissues revealed that *Glyma.19G193500* exhibited relatively high expression levels in stems, while *Glyma.19G193800* showed high expression in stems, roots, nodules, and flowers (Figure 5). Based on these findings, we speculate that these two genes may play important roles in stem development. Taken together, *Glyma.19G193500* (encoding a GDSL-type lipase) and *Glyma.19G193800* (encoding an AN1 zinc finger protein) are predicted to be candidate genes controlling soybean plant height and main stem node number.

## 3. Discussion

In this study, using two mixed soybean populations consisting primarily of germplasm from the Northwest production region but also including domestic and foreign accessions, we systematically analyzed the phenotypic variation in plant height and main stem node number. Through GWAS, we identified SNP loci on chromosome 19 significantly associated with both traits, and ultimately screened *Glyma.19G193500* (encoding a GDSL-type lipase) and *Glyma.19G193800* (encoding an AN1 zinc finger protein) as candidate genes.

### 3.1. Analysis of Phenotypic Variation in Plant Height and Main Stem Node Number

The interaction between plant genotype and different environments lead to variation in various agronomic traits. Studying the variation in different plant traits plays an important role in rapidly capturing key information related to the phenotypic characteristics of germplasm resources [22]. Previous studies have shown that the coefficients of variation for different agronomic traits in soybean differ considerably, with the coefficient of variation for plant height reaching up to 40.12%, while that for main stem node number is relatively smaller [23]. In this study, the average coefficient of variation for soybean plant height was 30.72%, and that for main stem node number was 23.675%. Compared with the results of Li Simeng et al. [24], the coefficient of variation for plant height was lower, while that for main stem node number was slightly higher or similar. This difference may be attributed to variations in population structure among different studies, such as differences in genetic background, environmental conditions, and sample size. Despite these differences, both traits exhibited relatively abundant genetic diversity in the population used in this study, indicating that this population harbors exploitable allelic variation and providing a solid phenotypic foundation for subsequent GWAS mapping and candidate gene mining.

### 3.2. GWAS Mapping Focused on Chromosome 19, Highlighting Its Central Role in Regulating Plant Architecture in Soybean

To reduce false positives caused by environmental and statistical model effects, this study adopted a strategy of joint detection across multiple populations and multiple environments. Ultimately, only 5 SNP loci associated with plant height and 18 SNP loci significantly associated with main stem node number were identified on chromosome 19. Notably, all SNP loci significantly associated with plant height were also significantly associated with main stem node number. This result is highly consistent with the findings of Wang et al. [17], who reported that loci associated with plant height and main stem node number are located on chromosome 19, further supporting the critical role of chromosome 19 in the genetic regulation of soybean plant height and main stem node number.

However, Zhang et al. [25] reported that loci associated with soybean plant height are distributed on chromosomes 3, 4, 6, and 19, showing some similarity to our findings. Nevertheless, our study detected associations only on chromosome 19 and not on other chromosomes, which may be due to the limited detection power of the models used for plant height, resulting in the failure to precisely identify certain loci. Despite this, the stable associated loci on chromosome 19 identified in this study provide reliable targets for subsequent fine mapping and gene functional studies.

### 3.3. Functional Prediction and Regulatory Mechanisms of Candidate Genes

The significance level is a core indicator for measuring the strength of association between each SNP locus and different traits, reflecting the reliability of the association, mapping accuracy, and locus stability. Extending 100 kb on each side of a significant SNP locus as a candidate interval and integrating gene expression data for candidate gene identification has become an important research strategy in GWAS [26,27,28]. Therefore, in this study, within the 100 kb upstream and downstream intervals flanking the major SNP loci, priority was given to genes harboring non-synonymous mutations. By integrating expression profile data, *Glyma.19G193500* and *Glyma.19G193800* were screened as candidate genes regulating plant height and main stem node number.

In this study, *Glyma.19G193500* was identified as encoding a GDSL-type lipase. Previous studies have shown that this class of lipases participates in the regulation of plant growth and development [29], in soybean, GDSL-type lipase family genes respond to drought stress, salt stress, and the abscisic acid (ABA) signaling pathway [30]. ABA, as a key hormone, can interact with gibberellins (GA), auxin, and other hormones to coordinately regulate cell elongation and internode development [31]. In this study, we found that this gene is highly expressed in stems. Therefore, we hypothesize that *Glyma.19G193500* may indirectly affect stem elongation and node formation in soybean by participating in the ABA signaling pathway.

*Glyma.19G193800* encodes an AN1 zinc finger protein belonging to the stress-associated protein family. In rice, the homologous gene OsDOG encodes a stress-associated protein that reduces gibberellin (GA) content by inhibiting GA activity and promoting GA degradation, thereby leading to shortened internodes and reduced plant height [32]. In soybean, stress-associated proteins have been demonstrated to participate in the ABA signaling pathway and affect plant stress tolerance [33]. In this study, this gene was found to be highly expressed in root tissues. Based on the above evidence, we hypothesize that *Glyma.19G193800* may regulate plant height and main stem node number through two pathways: first, by directly or indirectly affecting GA synthesis or degradation to alter GA levels; second, by interacting with other hormones via the ABA signaling pathway, ultimately influencing cell division and elongation. Notably, four SNP loci were found to be significantly associated with both plant height and main stem node number in this study, suggesting that these two traits may be regulated by the same genetic network, and the two candidate genes mentioned above may serve as important nodes within this network.

Plant growth and development depend on precise auxin levels and their spatial and temporal distribution. The recently proposed framework of hormone crosstalk further indicates that auxin not only independently regulates shoot morphogenesis but also coordinates environmental adaptability and plant architecture development through feedback loops with GA and ABA [34]. During normal plant growth and development, auxin promotes GA synthesis and acts antagonistically with ABA [35,36], while also interacting with other hormones to regulate their levels and functions, thereby affecting plant growth patterns. Therefore, any genetic variation that affects auxin synthesis, transport, or signal transduction may alter soybean plant height and main stem node number through the aforementioned hormone network. This mechanistic model also provides more complete theoretical support for understanding how the two candidate genes, *Glyma.19G193500* and *Glyma.19G193800*, in this study coordinately regulate plant height and node number through the hormone network.

In recent years, single-cell and spatial transcriptomics have provided powerful tools for dissecting the heterogeneity of different cell subpopulations and spatial expression gradients during plant development [37]. In future studies, gene functions can be validated at higher resolution by finely characterizing the cell-type-specific expression in stem and internode regions associated with candidate genes, combined with existing single-cell and spatial transcriptomic maps of soybean.

Although this study successfully identified SNP loci and candidate genes significantly associated with soybean plant height and main stem node number, providing important genetic resources for soybean breeding in Northwest China, the following limitations remain. (1) The significant SNP loci identified in this study are linked marker loci associated with the relevant traits. Only candidate genes harboring non-synonymous mutations in exon regions were retained, and phenotypic correlation analysis as well as gene expression difference analysis were performed. Since fine mapping and functional validation of the candidate genes have not been carried out, these loci cannot be determined as direct causal variants, and further experimental validation is required. (2) To improve screening efficiency, this study only focused on genes with non-synonymous mutations in exonic regions, without considering genes with regulatory region variants in introns or promoters. These genes may also be key genes controlling target traits. Subsequent analyses, such as interval variant interpretation and experimental validation, will be conducted to identify loci with regulatory region variants and to investigate whether the identified loci alter protein function in the coding region.

## 4. Materials and Methods

### 4.1. Plant Materials and Experimental Environments

The soybean germplasm resources required for this study are preserved in our laboratory, including a natural population of 384 Northwest soybean accessions (comprising 163 Northwest varieties, 105 foreign varieties, 8 unknown varieties, and 108 varieties from the Huang-Huai-Hai region, the Yangtze River region, the Northern spring soybean region, and the South China region) (Figure S1A), and a natural population of 372 Northwest soybean accessions (comprising 143 Northwest varieties and 229 foreign varieties) (Figure S1B). Moreover, 139 soybean accessions are shared between the two natural populations (Figure S2). The 372-accession natural population was planted in May 2024 in the experimental field of the Institute of Industrial Crops, Shanxi Agricultural University, in Fenyang City, Shanxi Province, and the 384-accession natural population was planted in May 2025 at the Dongyang Experimental Base in Yuci District, Shanxi Province. Each plot measured 2 m in length and 3 m in width. Within each plot, two soybean varieties were planted, with two rows per variety. The row spacing was 50 cm, and the plant spacing within rows was 13.5 cm. The spacing between different varieties within the same plot was 1 m.

### 4.2. Phenotypic and Statistical Analysis

After plant maturity, six plants with uniform growth were selected from each plot. Plant height was measured using a ruler from the cotyledon node to the highest point of the growing point, and main stem node number was recorded from the cotyledon node to the apex of the main stem. Phenotypic data for each agronomic trait were expressed as the average value of the six plants. Statistical analysis of the phenotypic data was performed using Microsoft Excel 2021 software, and normality tests were conducted using OriginPro 2024 software.

### 4.3. GWAS

Using SNP data obtained from previous resequencing of soybean germplasm in our laboratory, GWAS for soybean plant height and main stem node number was performed based on the R package (version 1.4.5) rMVP [38]. The MLM (Mixed Linear Model) model was employed. The Bonferroni method was used for multiple testing correction to control the false positive rate and to screen significant SNP loci [39]. A significance threshold of −log_10_(*p*) ≥ 5 was applied, and the association results were visualized using Manhattan plots and Q-Q plots.

### 4.4. Annotation and Expression Profiling of Candidate Genes

Using the PopLDdecay 3.43 software., the major locus was centered, and the upstream and downstream 100 kb regions were extracted for LD block analysis. Meanwhile, the upstream and downstream 100 kb regions flanking the physical position of the major SNP locus were defined as the search interval for candidate genes. To improve screening efficiency, priority was given to genes carrying non-synonymous SNP mutations in exonic regions, which were then compared against functional databases such as Pfam for functional annotation. Violin plots were drawn using GraphPad Prism 8 to analyze the correlation with phenotypic traits, and t-tests were performed to assess significant differences. Expression levels of candidate genes in different soybean tissues were downloaded from the Phytozome 14 online website (https://phytozome-next.jgi.doe.gov/, accessed on 18 August 2025), and heatmaps of expression levels were generated using TBtools 2024.1.11 software.

## 5. Conclusions

This study demonstrates that the natural soybean populations from Northwest China exhibit abundant genetic variation in plant height and main stem node number. Through GWAS, we identified a total of 5 SNPs significantly associated with soybean plant height and 18 SNPs significantly associated with main stem node number were identified. Among these loci, *Glyma.19G193500* (encoding a GDSL-type lipase) and *Glyma.19G193800* (encoding an AN1 zinc finger protein) are predicted to be candidate genes significantly associated with both plant height and main stem node number. These findings are expected to be applied in molecular marker-assisted breeding and gene editing breeding for the Northwest soybean production region, contributing to the development of new soybean varieties with high yield, stable yield, and broad adaptability, thereby positively impacting China’s soybean self-sufficiency rate and food security.

## Figures and Tables

**Table 1:** Variation analysis of plant height and main stem node number in soybean.

Location	Trait	Abbreviation	Average	SD	Minimum	Maximum	CV
Fenyang	Plant Height	FY_PH	84.21	22.55	25.00	144.2	26.78%
	Main Stem Node Number	FY_NN	13.79	3.16	5.60	21.40	22.91%
Dongyang	Plant Height	DY_PH	63.28	21.93	22.17	171.5	34.66%
	Main Stem Node Number	DY_NN	16.67	4.07	8.67	28.67	24.44%

**Table 2:** SNP loci significantly associated with plant height.

SNP	Chromosome	Position	−log_10_(*p*)	
			2024-Fenyang	2025-Dongyang
Gm19:48157078	19	48157078	5.716707	6.490348
Gm19:48157111	19	48157111	5.490156	5.63452
Gm19:48160670	19	48160670	5.576748	6.295255
Gm19:48168661	19	48168661	6.63809	6.32837
Gm19:48214573	19	48214573	6.624903	5.17713

**Table 3:** SNP loci significantly associated with node number on the main stem.

SNP	Chromosome	Position	−log_10_(*p*)	
			2024-Fenyang	2025-Dongyang
Gm19:48157078	19	48157078	7.625571	6.196078
Gm19:48157083	19	48157083	7.359354	6.174108
Gm19:48157111	19	48157111	7.726915	5.662312
Gm19:48160670	19	48160670	6.818454	6.240662
Gm19:48167999	19	48167999	5.688632	5.954577
Gm19:48168661	19	48168661	7.671296	6.064218
Gm19:48209702	19	48209702	9.016113	5.057276
Gm19:48210162	19	48210162	8.864907	5.049514
Gm19:48210932	19	48210932	8.89645	5.022246
Gm19:48211293	19	48211293	9.18984	5.019328
Gm19:48211710	19	48211710	6.329133	5.133076
Gm19:48212454	19	48212454	8.481549	5.019328
Gm19:48213085	19	48213085	8.345836	5.019328
Gm19:48214406	19	48214406	9.016113	5.29833
Gm19:48214573	19	48214573	8.89645	5.59979
Gm19:48216031	19	48216031	5.734818	5.09965
Gm19:48216495	19	48216495	7.916487	5.004287
Gm19:48217814	19	48217814	7.397611	5.174674

**Table 4:** Candidate genes associated with plant height on chromosome 19.

Gene ID	Functional Annotation
*Glyma.19G192800*	Alpha amylase, catalytic domain
*Glyma.19G192900*	ABC-transporter N-terminal
*Glyma.19G193100*	Protein kinase domain
*Glyma.19G193400*	bZIP transcription factor
*Glyma.19G193500*	GDSL-like Lipase
*Glyma.19G193800*	AN1-like Zinc finger
*Glyma.19G193900*	Calcineurin-like phosphoesterase
*Glyma.19G194100*	Protein LITTLE ZIPPER
*Glyma.19G194300*	Phosphatidylethanolamine-binding protein

**Table 5:** Candidate genes associated with number of main stem nodes on chromosome 19.

Gene ID	Functional Annotation
*Glyma.19G192800*	Alpha amylase, catalytic domain
*Glyma.19G192900*	ABC-transporter N-terminal
*Glyma.19G193100*	Protein kinase domain
*Glyma.19G193400*	bZIP transcription factor
*Glyma.19G193500*	GDSL-like Lipase
*Glyma.19G193800*	AN1-like Zinc finger
*Glyma.19G193900*	Calcineurin-like phosphoesterase
*Glyma.19G194100*	Protein LITTLE ZIPPER
*Glyma.19G194300*	Phosphatidylethanolamine-binding protein
*Glyma.19G194600*	F-box domain
*Glyma.19G194900*	Protein BIC

## Data Availability

The original contributions presented in this study are included in the article/Supplementary Materials. Further inquiries can be directed to the corresponding authors.

## Supplementary Materials

The following supporting information can be downloaded at: https://www.mdpi.com/article/10.3390/plants15111670/s1, Figure S1: Geographic distribution maps. (A) Geographic distribution of the 384 soybean accessions. (B) Geographic distribution of the 372 soybean accessions; Figure S2. Venn diagram showing the relationship between the two natural soybean populations from Northwest China; Table S1: Subgroup classification of 372 Northwest soybean natural populations and their plant height and main stem node number; Table S2: Subgroup classification of 384 Northwest soybean natural populations and their plant height and main stem node number; Table S3: SNP markers significantly associated with plant height and main stem node number by GWAS.

## Abbreviations

The following abbreviations are used in this manuscript:

GWAS	Genome-wide association analysis
SNP	Single nucleotide polymorphism
SD	Standard deviation
CV	Coefficient of variation
LD	Linkage disequilibrium
PH	Plant height
NN	Main stem node number
