# Supplementary material for: Genome-Wide Association Study and Candidate Gene Mining for Plant Height and Main Stem Node Number in Soybean from Northwest China
Source: Plants (Basel). 2026 May 29;15(11):1670. doi: 10.3390/plants15111670 (PMC13259219; doi:10.3390/plants15111670)
Supplement: Supplementary file 1 [file plants-15-01670-s001.zip › Supplementary Figures.pdf]

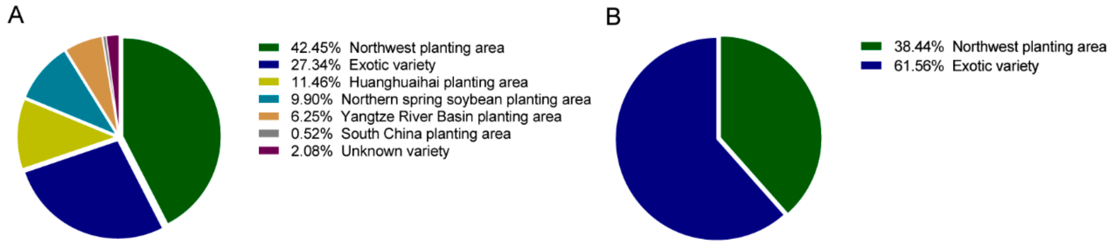

Figure S1. Geographic distribution maps. (A) Geographic distribution of the 384 soybean accessions. (B) Geographic distribution of the 372 soybean accessions.

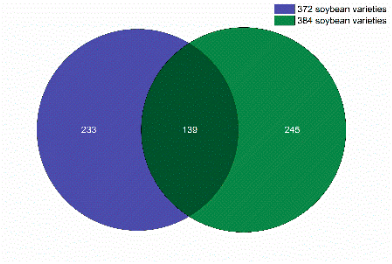

Figure S2. Venn diagram showing the relationship between the two natural soybean populations from Northwest China.
